# Association Between Gastroesophageal Reflux Disease and Chronic Respiratory Symptoms: A Systematic Review of Recent Clinical Evidence and Therapeutic Implications

**DOI:** 10.7759/cureus.91372

**Published:** 2025-08-31

**Authors:** Zeeshan Ahmed, Muhammad Habib Ur Rehman, FNU Abdul Rehman, Umair Arshad, Muhammad Zeeshan, Khaiber Khan, Mehwish Jabeen, Allahyar Khar

**Affiliations:** 1 Internal Medicine, King Edward Medical University, Lahore, PAK; 2 Internal Medicine, Services Institute of Medical Sciences, Lahore, PAK; 3 Internal Medicine, Sandeman Provincial Hospital, Quetta, PAK; 4 Internal Medicine, Nishtar Medical University, Multan, PAK

**Keywords:** asthma, chronic cough, copd, gastroesophageal reflux disease, laryngopharyngeal reflux, p-cab, ppi, respiratory symptoms, systematic review

## Abstract

Gastroesophageal reflux disease (GERD) is increasingly recognized as a potential contributor to chronic respiratory symptoms, yet its role in the pathogenesis and management of such conditions remains underexplored. This systematic review aimed to evaluate the association between GERD and respiratory manifestations including chronic cough, asthma, chronic rhinosinusitis, and chronic obstructive pulmonary disease (COPD). A comprehensive search was conducted across PubMed, Scopus, Web of Science, and Embase for studies published between January 2020 and April 2025. Six eligible studies, including randomized controlled trials and prospective observational studies, were analyzed. The findings demonstrate a consistent relationship between GERD and respiratory symptoms, with acid-suppressive therapies - particularly proton pump inhibitors (PPIs) and potassium-competitive acid blockers (P-CABs) - leading to symptomatic improvement in several patient populations. Non-pharmacological interventions such as diaphragmatic breathing also showed clinical benefit in GERD-induced chronic cough. Pediatric evidence, though limited, supported the role of GERD management in improving asthma control. Risk of bias across studies was generally low to moderate. While causality cannot be definitively established, the cumulative evidence highlights the importance of considering GERD in patients with refractory respiratory complaints. Integrating GERD assessment and treatment into respiratory care may enhance patient outcomes and reduce healthcare burden.

## Introduction and background

Gastroesophageal reflux disease (GERD) is a prevalent gastrointestinal disorder characterized by the retrograde movement of gastric contents into the esophagus, often resulting in troublesome symptoms such as heartburn, regurgitation, and chest discomfort [1]. Globally, GERD affects up to 20-30% of the population, with a rising trend observed due to lifestyle changes, increasing obesity, and dietary habits. While GERD is typically recognized for its esophageal manifestations, emerging evidence underscores its systemic impact, particularly on the respiratory tract [2,3].

The respiratory implications of GERD stem from two primary mechanisms: direct microaspiration of gastric contents into the airways and indirect vagally mediated reflexes triggered by esophageal acid exposure [4]. These mechanisms can induce inflammation, bronchoconstriction, and hypersensitivity of the respiratory mucosa. As a result, GERD has been increasingly associated with a spectrum of chronic respiratory conditions, including chronic cough, asthma, chronic obstructive pulmonary disease (COPD), laryngopharyngeal reflux (LPR), and chronic rhinosinusitis [5].

Chronic cough is one of the most frequently reported extra-esophageal symptoms of GERD, often persisting despite conventional respiratory treatment and only improving with anti-reflux therapy. Similarly, in asthma, particularly in patients with poorly controlled symptoms, GERD is thought to exacerbate bronchial hyperresponsiveness and impair pulmonary function [6]. In COPD, GERD may accelerate disease progression and increase the risk of acute exacerbations. Furthermore, conditions like LPR and chronic rhinosinusitis have been linked to upper airway inflammation, possibly triggered or aggravated by refluxed gastric contents.

Despite the growing interest in this field, the causal relationship between GERD and chronic respiratory symptoms remains a matter of debate. Many studies have explored this association, yet heterogeneity in study design, diagnostic criteria for GERD and respiratory conditions, and variability in outcome measurement limit definitive conclusions [7,8]. Understanding whether treating GERD improves respiratory symptoms is crucial for interdisciplinary management, especially in patients with overlapping gastrointestinal and respiratory complaints.

This systematic review aims to evaluate and synthesize current clinical evidence on the association between gastroesophageal reflux disease and chronic respiratory symptoms, focusing on studies that assess causality, clinical outcomes, and the impact of GERD-targeted interventions on respiratory health. By critically analyzing randomized controlled trials and clinical studies, this review seeks to clarify the extent and nature of the GERD-respiratory link and inform clinical practice and future research directions.

## Review

Materials and methods

Study Design and Protocol

This systematic review was conducted in accordance with the Preferred Reporting Items for Systematic Reviews and Meta-Analyses (PRISMA) 2020 guidelines [9]. The protocol was developed prior to data collection and adhered to a clearly defined PICO framework to structure the research question and guide the study selection process. The PICO model [10] was as follows: Population (P) - patients of any age with GERD; Intervention/Exposure (I) - presence or treatment of GERD (e.g., proton pump inhibitorss (PPIs), potassium-competitive acid blockers (P-CABs), diaphragmatic breathing); Comparison (C) - patients without GERD or receiving placebo/standard care; Outcome (O) - presence, improvement, or exacerbation of chronic respiratory symptoms, including chronic cough, asthma, COPD, LPR, and chronic rhinosinusitis.

Eligibility Criteria

Eligible studies were original research articles written in English and published in peer-reviewed journals within the last five years (2020-2025). Included studies had to report on the association between GERD and chronic respiratory symptoms or the impact of GERD-targeted interventions on such symptoms. Both adult and pediatric populations were considered. Studies were included if they were randomized controlled trials, clinical trials, or prospective observational studies with clearly defined diagnostic criteria for GERD and respiratory outcomes. Exclusion criteria included non-human studies, narrative reviews, editorials, letters, case reports, and studies lacking a clear respiratory endpoint or GERD diagnostic method.

Search Strategy

A structured literature search was conducted using PubMed, Scopus, Web of Science, and Embase to identify relevant studies. The search terms included combinations of Medical Subject Headings (MeSH) and free-text terms: ("Gastroesophageal Reflux" OR "GERD" OR "Acid Reflux") AND ("Chronic Cough" OR "Asthma" OR "COPD" OR "Chronic Rhinosinusitis" OR "Laryngopharyngeal Reflux" OR "Respiratory Symptoms") AND ("Treatment" OR "Association" OR "Outcome" OR "Randomized Controlled Trial"). The search was limited to human studies, English language, and articles published between January 2020 and April 2025. Titles and abstracts were screened independently by two reviewers, and the full texts of potentially eligible studies were retrieved for further assessment.

Study Selection and Data Extraction

From the identified records, six studies met the inclusion criteria and were selected for final analysis. These included four randomized controlled trials and two prospective observational studies. Data were independently extracted by two reviewers using a structured template. Extracted information included author name and year, study design, population characteristics, GERD diagnostic criteria, type of respiratory symptoms assessed, intervention/exposure details, comparison group (if applicable), and key findings related to GERD-respiratory outcomes.

Risk of Bias Assessment

The risk of bias for each included study was evaluated using standardized tools appropriate to the study design. The Cochrane Risk of Bias 2.0 (RoB 2.0) tool [11] was used for randomized controlled trials, while the Risk Of Bias In Non-randomized Studies - of Interventions (ROBINS-I) tool [12] was applied to assess the observational study. Each study was evaluated across domains such as randomization process, deviations from intended interventions, missing outcome data, outcome measurement, and selective reporting. The final judgment was categorized as low risk, some concerns, or high risk of bias, with disagreements resolved through discussion.

Data Synthesis

Given the heterogeneity in population characteristics, interventions, and outcome measures, a narrative synthesis was conducted instead of a meta-analysis. The findings were grouped and interpreted based on clinical outcomes, study quality, and intervention type (pharmacological vs. non-pharmacological). The synthesis focused on the presence and strength of the association between GERD and chronic respiratory symptoms, as well as the therapeutic implications of GERD-targeted interventions on respiratory health.

Results

Study Selection Process

**Figure 1:**
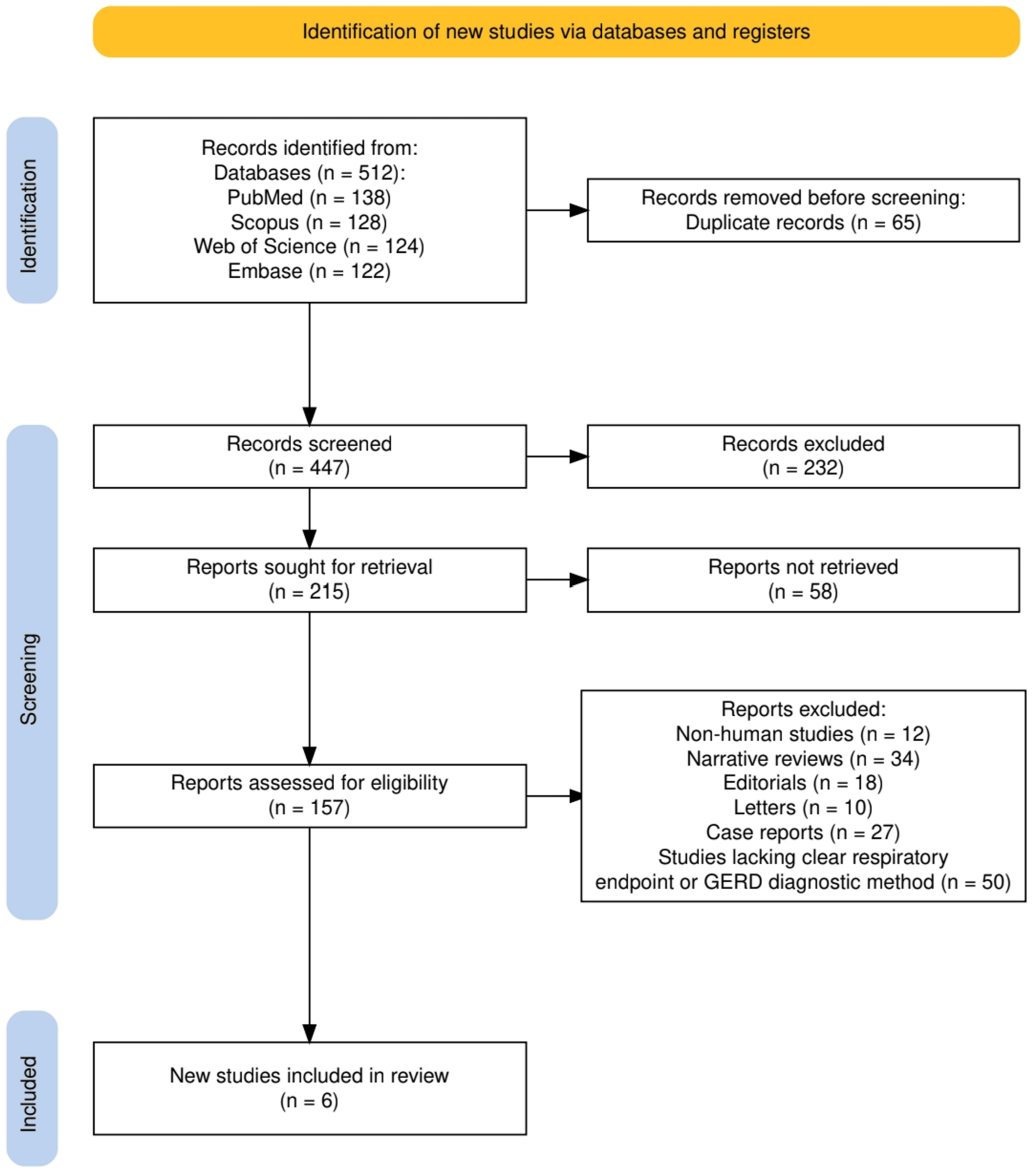
The PRISMA flow diagram represents the selection of the studies. PRISMA: Preferred Reporting Items for Systematic reviews and Meta-Analyses GERD: Gastroesophageal Reflux Disease

As illustrated in Figure 1, a total of 512 records were identified through database searches: PubMed (n = 138), Scopus (n = 128), Web of Science (n = 124), and Embase (n = 122). After the removal of 65 duplicates, 447 records remained for screening. Following title and abstract screening, 232 records were excluded. Of the 215 reports sought for retrieval, 58 could not be accessed. A total of 157 full-text articles were assessed for eligibility, and 151 were subsequently excluded for reasons including non-human studies (n = 12), narrative reviews (n = 34), editorials (n = 18), letters (n = 10), case reports (n = 27), and studies lacking a clear respiratory endpoint or a defined GERD diagnostic method (n = 50). Ultimately, six studies were included in the final review.

Characteristics of the Selected Studies

As summarized in Table 1, the six selected studies exhibit a diverse range of designs, populations, and respiratory outcomes in relation to GERD. The included studies comprised randomized controlled trials, double-blind clinical trials, and a large longitudinal observational study. Populations ranged from children with asthma to adults with chronic cough, laryngopharyngeal reflux disease (LPRD), COPD, and chronic rhinosinusitis. GERD diagnosis varied across studies, using tools such as Gastroesophageal Reflux Disease Questionnaire (GerdQ), pH monitoring, symptom indices, and esogastroscopy. Interventions predominantly involved PPIs, H2 blockers, or novel agents like fexuprazan and were compared either with standard treatments or control groups. Key findings across the studies consistently highlighted a positive association between GERD treatment and improvement in respiratory outcomes, including cough resolution, asthma control, symptom severity, and lung function preservation. Notably, some studies also emphasized specific subgroups, such as children or patients with severe baseline symptoms, showing more pronounced benefits from GERD-targeted interventions.

**Table 1:** The characteristics of the included studies in the review. RCT: Randomized Controlled Trial QoL: Quality of Life LCQ: Leicester Cough Questionnaire NRS: Numerical Rating Scale OR: Odds Ratio GERD: Gastroesophageal Reflux Disease GERC: Gastroesophageal Reflux-induced Chronic Cough LPRD: Laryngopharyngeal Reflux Disease CRSnNP: Chronic Rhinosinusitis without Nasal Polyps COPD: Chronic Obstructive Pulmonary Disease FEV1: Forced Expiratory Volume in 1 Second FVC: Forced Vital Capacity GerdQ: Gastroesophageal Reflux Disease Questionnaire RSI: Reflux Symptom Index RFS: Reflux Finding Score C-ACT: Childhood Asthma Control Test GERD-HRQL: GERD Health-Related Quality of Life Questionnaire QCT: Quantitative Computed Tomography FESS: Functional Endoscopic Sinus Surgery DEP: Deep Diaphragmatic Breathing PPI: Proton Pump Inhibitor H2 blockers: Histamine-2 Receptor Antagonists Fexuprazan: Potassium-Competitive Acid Blocker (P-CAB) Esomeprazole: Proton Pump Inhibitor Omeprazole: Proton Pump Inhibitor pH monitoring: Measurement of acidity in the esophagus using a probe

Study (Author, Year)	Study Design	Population	GERD Definition / Diagnosis	Respiratory Outcome(s)	Intervention / Exposure	Comparison	Key Findings
Niu et al., 2024 [13]	Randomized Controlled Trial	60 adults with GERD-induced chronic cough; 30 per group	Diagnosis of GERC; evaluated using GerdQ and clinical criteria	Chronic cough (assessed by symptom scores, LCQ), cough sensitivity, sleep quality	Routine GERD treatment + Deep diaphragmatic breathing (DEP) training	Routine GERD treatment only (control group)	DEP group showed significantly higher cough resolution (94% vs. 77%), improved GerdQ, LCQ, sleep quality, anxiety/depression scales, and increased diaphragmatic muscle activity
Kang et al., 2025 [14]	Randomized, Double-Blind, Active-Controlled Trial	161 adults with chronic cough ≥8 weeks and recent GERD/peptic symptom diagnosis; 146 completed the trial	Recent physician-diagnosed GERD or peptic symptoms (within <1 month); symptom-based diagnosis	Chronic cough (assessed by LCQ, NRS), cough-related QoL	Fexuprazan 40 mg once daily for 8 weeks	Esomeprazole 40 mg once daily for 8 weeks	Both groups showed significant improvement in LCQ scores; no significant difference between Fexuprazan and Esomeprazole groups. Similar outcomes in cough severity and RDQ scores. Mild adverse events in both groups
Yagoubi et al., 2022 [15]	Randomized Controlled Trial	102 children (aged 4–16) with poorly controlled asthma; 59 diagnosed with GERD via pH monitoring	GERD diagnosed by 24-hour pH monitoring	Asthma control assessed via the Childhood Asthma Control Test (C-ACT)	Omeprazole for 6 months in GERD+ group	No GERD treatment in GERD+ control group	Omeprazole significantly improved asthma control in children with GERD (84.8% improved vs. 11.5%, p
Baldomero et al., 2020 [16]	Longitudinal Observational Study (Clinical Trial – COPDGene Cohort)	5728 COPD patients from COPDGene study; 5-year follow-up between Phase I and II	Self-reported physician-diagnosed GERD	Lung function decline (FEV1, FVC), QCT measures (air trapping, airway wall thickness, emphysema)	Presence of GERD ± treatment (PPI/H2 blockers)	COPD patients without GERD	GERD was associated with higher odds of rapid FEV1 decline (OR 1.20), and greater progression of QCT-measured air trapping. PPI/H2 blocker use was linked to faster decline in FEV1 and FVC among GERD patients
Kim et al., 2024 [17]	Randomized, Double-Blind, Multicenter Clinical Trial	136 adult patients with LPRD; RSI ≥13 and RFS ≥7	Diagnosed by Reflux Symptom Index (RSI) and Reflux Finding Score (RFS)	LPR-related symptoms: hoarseness, troublesome cough (assessed via RSI and symptom questionnaires)	Fexuprazan 40 mg daily for 8 weeks	Esomeprazole 40 mg daily for 8 weeks	Both groups showed significant symptom improvement; in patients with severe symptoms (RSI ≥18), fexuprazan led to faster improvement in hoarseness and cough compared to esomeprazole (p = .036 and .045)
Lechien et al., 2021 [18]	Randomized Controlled Trial with 10-Year Follow-Up	57 adult patients with chronic rhinosinusitis without nasal polyps (CRSnNP)	GERD assessed via GERD-HRQL questionnaire; confirmed by esogastroscopy in symptomatic patients	Chronic rhinosinusitis recurrence, treatment resistance, need for FESS	GERD presence and severity in CRSnNP patients	CRSnNP patients without GERD symptoms	GERD severity was associated with late recurrence of CRSnNP and reduced treatment response; suggests GERD may negatively influence long-term sinus outcomes

Quality Assessment

As shown in Table 2, the quality assessment revealed that most randomized controlled trials were judged to have a low risk of bias, with well-described randomization processes, appropriate blinding, validated outcome measures, and minimal missing data. However, some studies raised concerns, particularly due to unclear allocation concealment, lack of detailed blinding procedures, or reliance on subjective assessments. The single observational study included showed some risk of bias primarily due to the use of self-reported diagnoses and potential confounding factors, despite efforts to address them. Additionally, studies with extended follow-up durations introduced possible attrition and detection bias. Overall, while the methodological quality was generally acceptable, certain limitations in design and reporting highlight the need for cautious interpretation of results.

**Table 2:** The risk of bias assessment of the included studies. RCT: Randomized Controlled Trial RoB: Risk of Bias RoB 2.0: Revised Cochrane Risk of Bias Tool for Randomized Trials (version 2.0) ROBINS-I: Risk Of Bias In Non-randomized Studies - of Interventions GERD: Gastroesophageal Reflux Disease

Study (Author, Year)	Study Design	RoB Tool Used	Risk of Bias Judgment	Justification
Niu et al., 2024 [13]	Randomized Controlled Trial	RoB 2.0 (Cochrane tool for RCTs)	Low Risk	Well-described randomization; outcome measures clearly defined; balanced groups; minimal missing data
Kang et al., 2025 [14]	Randomized, Double-Blind RCT	RoB 2.0	Low Risk	Double-blind, active-controlled design; appropriate statistical handling; minimal attrition bias
Yagoubi et al., 2022 [15]	Randomized Controlled Trial	RoB 2.0	Some Concerns	Allocation concealment and blinding not clearly described; objective outcome but observer bias possible
Baldomero et al., 2020 [16]	Longitudinal Observational Study	ROBINS-I	Some Concerns	GERD was self-reported (risk of misclassification); confounding addressed but not fully eliminated
Kim et al., 2024 [17]	Randomized, Double-Blind RCT	RoB 2.0	Low Risk	Adequate blinding and allocation; validated symptom scores; pre-registered trial
Lechien et al., 2021 [18]	Randomized Controlled Trial with 10-Year Follow-Up	RoB 2.0	Some Concerns	Long-term follow-up may introduce attrition bias; GERD diagnosis partially subjective; retrospective GERD assessment could introduce detection bias

Discussion

Our systematic review synthesized evidence from six clinical studies exploring the association between GERD and chronic respiratory symptoms. Across diverse populations - including adults with chronic cough, children with asthma, patients with chronic rhinosinusitis, and those with COPD - a consistent link emerged between GERD and respiratory manifestations. Chronic cough was the most frequently studied symptom, with multiple trials demonstrating improvement following acid suppression therapy using either PPIs or P-CABs. Notably, non-pharmacological interventions such as deep diaphragmatic breathing also showed substantial benefit, as evidenced in Niu et al.’s study [13], where 94% of participants experienced cough resolution compared to 77% in the control group. Pediatric data from Yagoubi et al. [15] further supported this association, showing significantly improved asthma control with omeprazole in children with pH-proven GERD. Meanwhile, studies involving adults with COPD and chronic rhinosinusitis suggested that GERD may accelerate disease progression or hinder treatment response. The findings spanned both randomized controlled trials and longitudinal cohort studies, with low to moderate risk of bias, further strengthening the credibility of the observed associations.

These results align with existing theories and clinical observations suggesting a multifactorial link between GERD and respiratory pathology. The most plausible mechanisms include microaspiration of gastric contents leading to airway inflammation, as well as vagally mediated esophageal-bronchial reflexes that induce bronchoconstriction in response to acid exposure in the distal esophagus [19]. These theories are reinforced by the consistent improvement in cough-related quality of life and asthma control following GERD-targeted interventions in the reviewed studies. For instance, the comparable efficacy of fexuprazan and esomeprazole in treating GERD-related chronic cough highlights the importance of acid suppression irrespective of drug class, while the COPDGene cohort demonstrated measurable structural lung changes associated with self-reported GERD [14]. Although causality cannot be definitively confirmed in all cases, the convergence of data from pharmacological and non-pharmacological interventions across both pediatric and adult populations adds weight to the hypothesis that GERD is not merely a coexisting condition, but a potential contributor to chronic respiratory morbidity [20].

An important insight emerging from this review is the underappreciated diagnostic role of GERD in patients presenting with chronic, unexplained respiratory symptoms. GERD often remains clinically silent or is overlooked when evaluating respiratory conditions like chronic cough, asthma, or chronic rhinosinusitis. Notably, interventions aimed at managing GERD - including both pharmacologic options like PPIs and P-CABs, and non-pharmacologic strategies such as diaphragmatic breathing - demonstrated measurable respiratory improvement even when traditional respiratory therapies had limited effect [21]. The utility of P-CABs, particularly fexuprazan, in rapidly alleviating laryngopharyngeal symptoms such as hoarseness and cough suggests a pharmacological advantage potentially rooted in faster onset and greater acid suppression stability [22]. Furthermore, this review highlights the critical paucity of longitudinal pediatric data, despite the compelling findings from Yagoubi et al. [15], where GERD-directed treatment significantly improved asthma control in children. These observations advocate for a broader clinical suspicion and more integrative GERD screening, especially in patients with refractory respiratory complaints.

Despite the strength of evidence gathered, certain limitations must be acknowledged. At the study level, several trials relied heavily on subjective symptom questionnaires without objective physiological assessments such as esophageal pH monitoring or impedance testing, which may lead to misclassification of GERD severity or type (acid vs. non-acid). Sample sizes in some trials, such as those by Niu et al. and Lechien et al., were relatively small, which may affect statistical power and generalizability. Additionally, while most included studies were randomized controlled trials, not all were blinded or free from performance bias, as reflected in our risk of bias assessment. At the review level, significant heterogeneity in study design, respiratory outcomes assessed, and GERD diagnostic criteria precluded the possibility of meta-analysis. This underscores the methodological variability that currently challenges robust synthesis and may limit direct comparisons across studies.

The findings of this review carry meaningful clinical implications, especially for primary care, pulmonology, and gastroenterology practice. Clinicians should maintain a high index of suspicion for GERD in patients with chronic cough, poorly controlled asthma, or unexplained upper airway symptoms, particularly when conventional respiratory treatments yield suboptimal results [23]. Screening tools such as the GerdQ or Reflux Symptom Index (RSI)/Reflux Finding Score (RFS) can provide a practical starting point for identifying patients who may benefit from acid suppression therapy [24,25]. Moreover, in patients with COPD, even a mild history of GERD may signal the potential for faster pulmonary decline, warranting more vigilant monitoring. Non-pharmacologic interventions like diaphragmatic breathing training also warrant consideration as adjunctive strategies, particularly in individuals with functional respiratory complaints or those seeking drug-free alternatives. Ultimately, an integrated, multidisciplinary approach to GERD-respiratory symptom management can enhance both diagnostic accuracy and therapeutic outcomes.

This review also exposes several key gaps in the existing literature that require urgent attention. Most notably, there is a lack of large-scale, double-blind randomized trials specifically designed to evaluate respiratory outcomes as primary endpoints in GERD management. Standardization of GERD diagnostic criteria across respiratory-focused studies is also necessary to improve comparability and reproducibility. Furthermore, the role of non-acid or weakly acidic reflux remains underexplored, particularly in patients who remain symptomatic despite optimal PPI therapy - an area where impedance-pH monitoring may provide more accurate assessment [26]. The interaction between GERD and treatment resistance in conditions like chronic rhinosinusitis or LPR also remains poorly defined and deserves further exploration, especially regarding how reflux modifies local inflammation and mucosal sensitivity. Future research should also seek to evaluate long-term outcomes of combined pharmacologic and non-pharmacologic interventions and include more pediatric and elderly populations to understand age-related variations in disease manifestation and response.

## Conclusions

This systematic review underscores a clinically meaningful association between gastroesophageal reflux disease and a range of chronic respiratory symptoms, most notably chronic cough, asthma, and upper airway conditions. Acid suppression therapies and breathing-based interventions demonstrated tangible respiratory benefits, reinforcing the need to view GERD as more than a gastrointestinal disorder. Recognizing and managing GERD in patients with respiratory symptoms may not only alleviate their burden but also reduce unnecessary investigations and enhance overall care. The incorporation of reflux assessment into respiratory diagnostic protocols, especially in refractory cases, can be a crucial step forward in improving patient outcomes through a multidisciplinary lens.
